# The accumulation of plasma acylcarnitines are associated with poor immune recovery in HIV-infected individuals

**DOI:** 10.1186/s12879-021-06525-6

**Published:** 2021-08-12

**Authors:** Shi Qian, Xi Chen, Tong Wu, Yu Sun, Xiaolin Li, Yajing Fu, Zining Zhang, Junjie Xu, Xiaoxu Han, Haibo Ding, Yongjun Jiang

**Affiliations:** 1 NHC Key Laboratory of AIDS Immunology (China Medical University), National Clinical Research Center for Laboratory Medicine, The First Affiliated Hospital of China Medical University, Shenyang, 110001 China; 2 Department of Clinical Laboratory, Tianjin Medical University General Hospital, Tianjin, 300052 China; 3 Department of Clinical Laboratory, The Affiliated Hospital of Qingdao University, Qingdao, 266000 China

**Keywords:** HIV, ART, Immune recovery, Metabolomics, Biomarkers

## Abstract

**Background:**

Antiretroviral therapy (ART) can reduce opportunistic infections and mortality rates among individuals infected with human immunodeficiency virus (HIV); however, some HIV-infected individuals exhibit poor immune recovery after ART. Hence, we explored the association between metabolome profiles and immune recovery in HIV-infected individuals following ART.

**Methods:**

An untargeted metabolomics approach was used to analyze plasma samples from 18 HIV-negative individuals and 20 HIV-infected individuals, including 10 immunological non-responders (INR, CD4^+^ T cell rise < 100 cells/μl) and 10 immunological responders (IR, CD4^+^ T cell rise > 300 cells/μl) after 2 years of ART. These individuals were followed for the next 6 years and viral loads and CD4^+^ T cell count were measured regularly. Orthogonal projection on latent structures discriminant analysis (OPLS-DA), ANOVA, correlation, receiver operating characteristic (ROC), and survival analyses were used for selection of discriminant metabolites.

**Results:**

Eighteen lipid metabolites were identified which could distinguish among control, INR, and IR groups. Among them, myristoylcarnitine (MC), palmitoylcarnitine (PC), stearoylcarnitine (SC), and oleoylcarnitine (OC) were significantly elevated in INR plasma samples compared with those from the IR and control groups and were negatively associated with CD4^+^ T cell count. Additionally, ROC analysis using a combination of MC, PC, SC, and OC had high sensitivity and specificity for differentiating INR from IR (AUC = 0.94). Finally, survival analysis for the combination of MC, PC, SC, and OC demonstrated that it could predict CD4^+^ T cell count in patients undergoing long-term ART.

**Conclusions:**

High levels of lipid metabolites, MC, PC, SC, and OC are associated with poor immune recovery in patients receiving ART and these data provide potential new insights into immune recovery mechanisms.

## Background

Antiretroviral therapy (ART) can suppress plasma viral RNA to undetectable levels, promote immune recovery, and efficiently reduce opportunistic infections and mortality rates among individuals infected with human immunodeficiency virus (HIV) [1–4]; however, some HIV-infected individuals, referred to as “immunological non-responders” (INR) have poor immune recovery after ART, and are at increased risk of rapid disease progression and death [5]. Poor immune recovery has been associated with sex, baseline CD4^+^ and CD8^+^ T cell count, and HIV RNA levels, as well as related to alterations in T cell phenotype, function, and PD-1 expression. Further, recent reports have demonstrated that various metabolites, such as serine and polysaccharides, can affect the proliferation and immune function of CD4^+^ T cells in healthy people [6–9].

Metabolomics is a powerful discovery tool for exploration of biomarkers and potential disease mechanisms, in conditions such as liver disease, cardiovascular disease, prostate cancer, obesity, and diabetes. A recent study measured lipoproteins by nuclear magnetic resonance and found that HIV-infected patients with high levels of large HDL particles, HDL cholesterol, and larger sized LDL particles had superior immunological recovery after treatment [10]. In addition, a Spanish research team investigated the metabolic characteristics of gut bacteria in immunological responders (IR) and INR, demonstrating that the gut microbiome interacts with immune recovery [11]. Nevertheless, limited information is available regarding the association of plasma metabolite profiles with immune recovery in HIV-infected individuals following ART. Here, we attempted to discover associations between metabolites and immune recovery, and to identify biomarkers which could predict CD4^+^ T cell count during continuous treatment and after ART in HIV-infected individuals, using a metabolomic approach.

In this study, we describe the plasma metabolite profiles of controls, IR, and INR, detected by ultra-high-performance liquid chromatography-tandem mass spectrometry (UPLC-MS/MS). We demonstrate associations of myristoylcarnitine (MC), palmitoylcarnitine (PC), stearoylcarnitine (SC), and oleoylcarnitine (OC) with poor immune recovery in individuals receiving ART and found that levels of these lipids could predict CD4^+^ T cell count in later treatment.

## Methods

### Study subjects

All subjects enrolled were men who have sex with men recruited from the First Affiliated Hospital of China Medical University and provided written informed consent. The study protocol was approved by the Research and Ethics Committee of The First Affiliated Hospital of China Medical University, in compliance with the Declaration of Helsinki. Twenty ART-treated HIV-infected individuals, including 10 INR and 10 IR, where INR and IR were defined by a CD4^+^ T cell count rise after 2 years of < 100 or > 300 cells/μl of ART (viral loads < 20 copies/ml) were collected from the Red Ribbon Clinic. Treatment regimens comprised nucleoside reverse transcriptase inhibitors (NRTI) plus non-NRTI (TDF/AZT + 3TC + EFV/NVP). Subjects selected for inclusion did not have any laboratory abnormalities related to glucose, or kidney or liver function measurements, and were not co-infected with hepatitis, tuberculosis, syphilis, or other infectious diseases. In addition, 18 HIV-negative individuals were used as healthy controls. Age and body mass index (BMI) did not differ significantly among three groups. Participant characteristics are presented in Table 1.

**Table 1:** Characteristics of subjects enrolled in this study

Characteristics	Controls	INR	IR	*p*-value (IR vs INR)
Number of subjects	18	10	10	
Male (%)	100	100	100	
Age (years)	37 (35, 38)	33 (29, 49)	39 (30, 43)	> 0.05
Body mass index (kg/m^2^)	23 (20.3, 23.0)	21.7 (20.6, 22.8)	21.5 (20.9, 21.9)	> 0.05
Glucose (mmol/L)	NA	5.3 (4.5, 5.6)	5.0 (4.9, 5.4)	> 0.05
AST (U/L)	NA	26.5 (23.8, 30.2)	24.6 (23.0, 37.0)	> 0.05
ALT (U/L)	NA	26.5 (20.3, 35.0)	31.0 (19.0, 50.0)	> 0.05
Triglyceride (mmol/L)	NA	1.23 (1.00, 1.33)	1.28 (1.09, 1.79)	> 0.05
Total cholesterol (mmol/L)	NA	3.44 (3.36, 3.90)	4.06 (3.56, 4.28)	> 0.05
Viral load (copies/mL)	NA	< 20	< 20	
CD4^+^ T cell count (cells/μL)				
Baseline	NA	162 (95, 179)	97 (79, 154)	> 0.05
At 2 years of ART	NA	219 (163, 234)	456 (383, 510)	< 0.05
Drug regimen				
EFV + 3TC + AZT	NA	2	1	> 0.05
EFV + 3TC + TDF	NA	1	1	
NVP + 3TC + AZT	NA	7	8	

Data are expressed as the median (interquartile range), unless otherwise stated. *NA* not available, *INR* immunological non-responders, *IR* immunological responders, *AST* aspartate aminotransferase, *ALT* alanine aminotransferase

### HIV viral load test

Plasma samples were prepared and analyzed using the COBAS® TaqMan® system with COBAS® AmpliPrep/COBAS® TaqMan® HIV-1 Test Kits v2.0 (Roche Molecular Systems, USA). The upper and lower detection limits of the kit were 10^7^ copies/mL and 20 copies/mL, respectively.

### Detection of CD4^+^ T cell count

Fresh anticoagulant blood was stained with TriTEST anti-CD4-FITC/CD8-PE/CD3-PerCP reagent in Trucount tubes (BD Biosciences, San Jose, CA, USA). Then, hemolysin was added to lyse the red blood cells. CD4^+^ T cells were detected and enumerated using a FACS Calibur flow cytometer (BD Biosciences, San Jose, CA, USA).

### Metabolomic profiling

Venous blood samples were drawn after an overnight fasting. Plasma samples were obtained and assigned a unique identifier using a laboratory information management system, and kept at -80℃ until processed. An automated MicroLab STAR® system (Hamilton Company, Reno, NV, U.S.A) was used to prepare samples, as described previously [12, 13]. Briefly, several recovery standards were added before extraction for quality control purposes; proteins were precipitated in methanol by vigorous shaking, followed by centrifugation to remove proteins; the resulting extracts were divided into five parts, of which four were analyzed using different methods and one was retained as a backup. Samples were placed momently on a TurboVap® (Zymark, Westborough, MA, USA) to deplete the organic solvent, then stored overnight in nitrogen before analysis.

Detailed descriptions of non-targeted metabolomics analysis can be found in published work [13]. In summary, sample extracts were dried and resuspended in specific solvents for each of four methods: one aliquot was analyzed by hydrophilic interaction liquid chromatography/UPLC-MS/MS, with negative ion mode electrospray ionization (ESI); two aliquots were analyzed using two separate reverse phases (RP)/UPLC-MS/MS methods, with positive ion mode ESI; and the final aliquot was analyzed by RP/UPLC-MS/MS with negative ion mode ESI. All methods were conducted using a Waters ACQUITY UPLC and a Thermo Scientific Q-Exactive high resolution/accurate mass spectrometer, which were coupled with a heated electrospray ionization (HESI-II) source and an Orbitrap mass analyzer, with a working mass resolution of 35,000. The scanning range varied slightly between different methods but covered 70–1000 m/z. Raw data files were extracted and archived.

Data extraction and compound identification procedures were as previously described [14]. Briefly, Metabolon software and hardware were used to extract the original data, identify peak values, and carry out quality control processes, while proprietary visualization and interpretation software were used to confirm peak identification.

### Data processing, bioinformatics, and statistical analysis

For metabolomics data, variables with > 20% missing values were excluded [15]. For the remaining variables, missing values were replaced with the lower limit of detection. Metabolite data detected across multiple days were normalized by setting the medians to equal one and then normalizing each data point proportionately. The multivariate statistical methods, orthogonal partial least square discriminant analysis (OPLS-DA) was conducted using SIMCA 14.1 (Umetrics, Sweden). One-way ANOVA with Fisher’s LSD test was performed for comparisons of variables among the three groups, and the Benjamini and Hochberg false discovery rate (FDR) method was used for corrections, with a cut-off level of 5%. A heatmap of the 30 metabolites that differed significantly among the three groups was produced using MultiExperiment Viewer 4.9.0 [16]. Fold-change values of A/B were calculated as the ratios of mean levels of metabolites in group A to those in group B. Metabolite Set Enrichment Analysis were performed using MetaboAnalyst 4.0 [17].Correlation analysis was performed using the Spearman correlation test. Receiver operating characteristic (ROC) analysis was used to assess ability to predict immune recovery. For ROC analysis of the four-metabolite combination, predicted probability was calculated for inclusion in the ROC analysis by binary logistic regression. Then, the best diagnostic thresholds for metabolites, with the highest sensitivity and specificity values, were selected and used to regroup the 20 HIV-infected subjects, followed by Kaplan–Meier survival analysis plus a log-rank test to evaluate the influence of potential biomarkers on CD4^+^ T cell count. Statistical analyses were conducted using R (http://cran.r-project.org/) and GraphPad Prism 8.0.2. Two-sided test *p* values < 0.05 were considered significant.

## Results

### Overall metabolic signatures of plasma samples

An untargeted metabolomics assay was performed to detect plasma metabolites in controls, IR and INR after 2 years of ART. A total of 330 known compounds were identified. To reduce noise in the analysis, variables with > 20% missing values and xenobiotics were excluded. The remaining 241 metabolites included 125 lipids (52%), 68 amino acids (28%), 7 peptides (3%), 14 carbohydrates (6%), 12 cofactors and vitamins (5%), 9 nucleotides (4%), and 6 energy metabolites (2%).

**Fig. 1:**
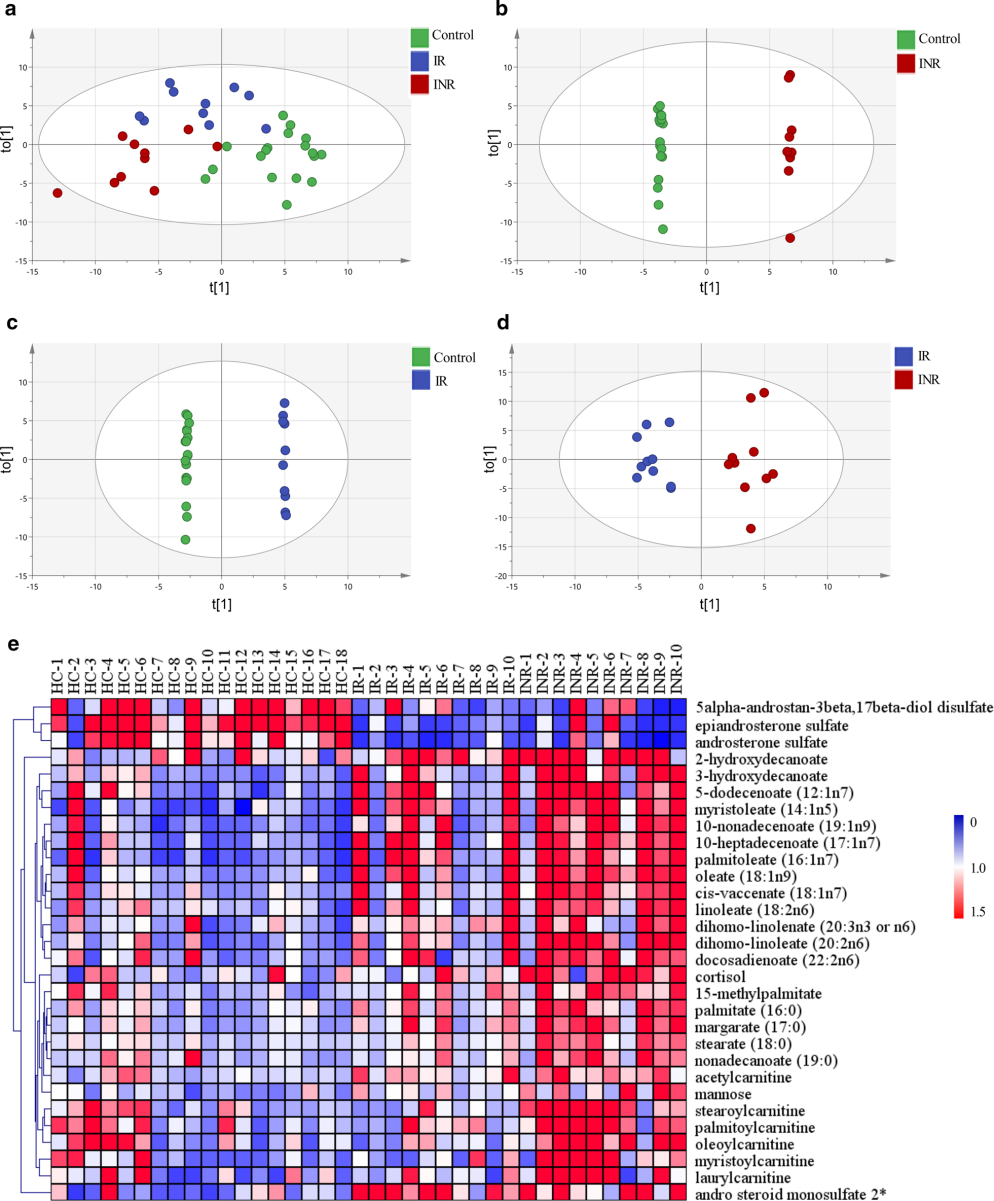
Metabolite profiles in the control, INR, and IR groups.** a**–**d** OPLS-DA score plots derived from untargeted metabolism profiles of plasma samples. **e** Hierarchical cluster analysis and heat map visualization of the top 30 variables ranked by ANOVA

OPLS-DA, a supervised clustering method, was applied to determine which metabolites significantly contributed to the observed separation among groups. All metabolites were used to build OPLS-DA models and model parameters are presented in Table 2. The score plot showed good separation among the three groups (INR, IR, and controls), with R^2^Y = 0.782, and Q^2^ (cum) = 0.184, indicating a good capacity for fitting and prediction (Fig. 1a). Since OPLS-DA may lead to over-fitted models, a permutation test (999 iterations) was performed and the Q^2^ intercept value was -0.294, indicating that the model was not overfitted.

**Table 2:** Parameters for OPLS-DA models

Model	OPLS-DA models				Permutation	
	Components^#^	R^2^X (cum)	R^2^Y (cum)	Q^2^ (cum)	R^2^ intercept	Q^2^ intercept
Controls vs IR vs INR	2P + 1O	0.250	0.782	0.184	0.513	− 0.294
Controls vs INR	1P + 4O	0.408	0.999	0.763	0.994	− 0.249
Controls vs IR	1P + 5O	0.421	1.000	0.462	0.999	− 0.142
IR vs INR	1P + 1O	0.134	0.601	0.076	0.936	− 0.017

^#^*P* predictive, *O* orthogonal

Furthermore, OPLS-DA models for binary group comparisons were generated and variable influence in projection (VIP) values were calculated. For control and INR samples, the model had 1 predictive and 4 orthogonal components, and its validation parameters were R^2^Y = 0.999, and Q^2^ (cum) = 0.763 (Fig. 1b). For control and IR samples, the model had 1 predictive and 5 orthogonal components, and its validation parameters were R^2^Y = 1.000, and Q^2^ (cum) = 0.462 (Fig. 1c). For IR and INR samples, the model had 1 predictive and 1 orthogonal component, and its validation parameters were R^2^Y = 0.601, and Q^2^ (cum) = 0.076 (Fig. 1d). In all these models, permutation tests (999 iterations) were conducted, and all resulting Q^2^ intercept values were negative, indicating that the models were not overfitted. VIP scores > 2 were considered to indicate variables contributing to the separation of groups.

### Identification of metabolites which could distinguish IR from INR metabolomics profiles

To select potential biomarkers of immune recovery, metabolites were identified which differed significantly among the three groups and between IR and INR with a *p* value < 0.05 (FDR < 5%) based on the results of ANOVA. We found that the levels of 30 metabolites differed significantly among the three groups: most were lipids, all differed between INR and control samples, 14 differed between IR and control samples, and 16 between IR and INR. The heatmap depicts levels of these 30 metabolites and shows two main variable clusters (Fig. 1e). The levels of some steroids were decreased in IR and INR compared with control samples, while high levels of fatty acids and acylcarnitines were detected in INR samples compared with control and IR samples.

**Fig. 2:**
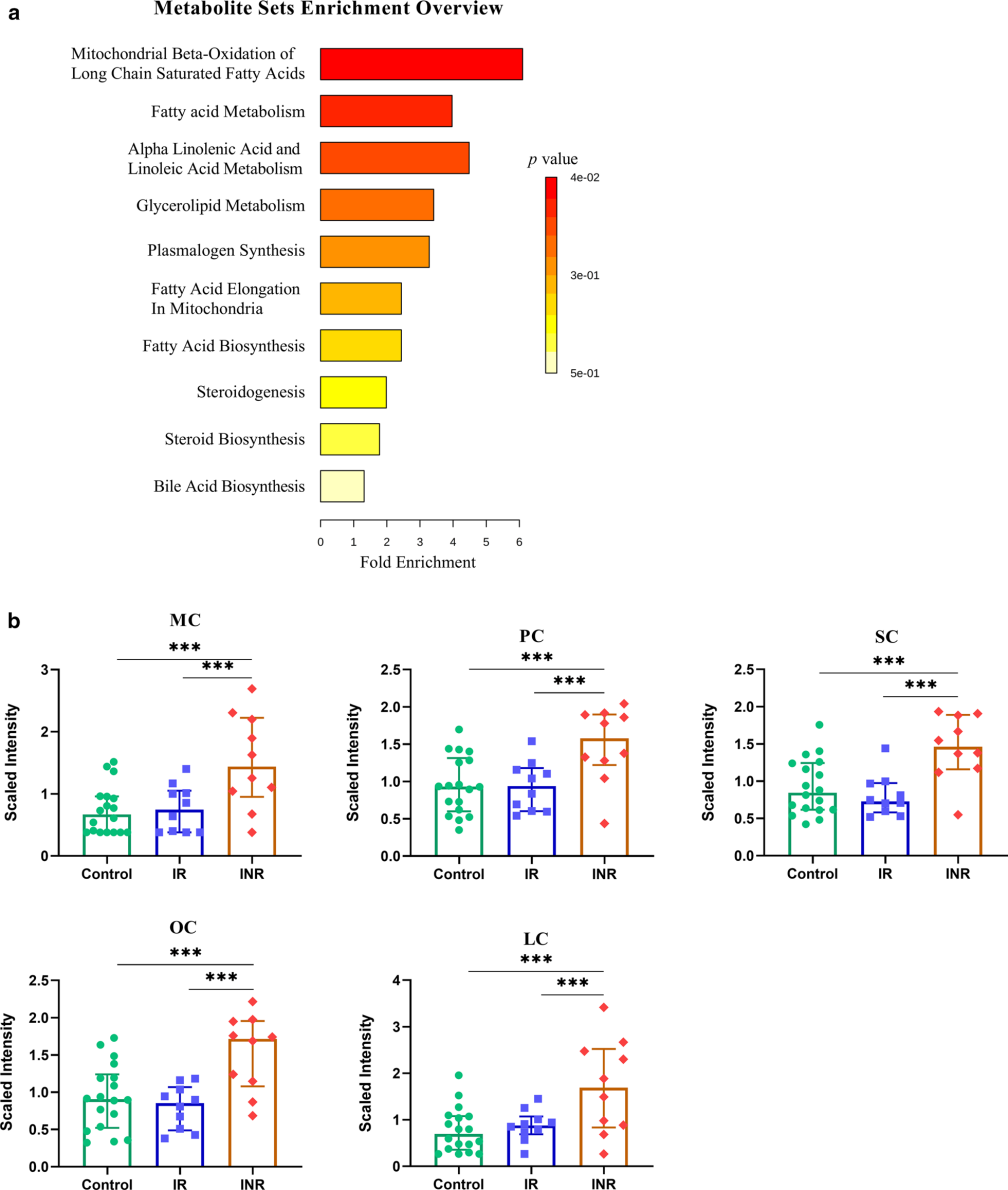
Acylcarnitines were identified as the metabolites exhibiting the main differences between IR and INR. **a** Metabolite Set Enrichment Analysis using 18 metabolites which could distinguish controls, IR, and INR. **b** Box-and-whisker plots of the metabolite levels in control (n = 18), IR (n = 10), and INR (n = 10) samples. ANOVA with Fisher’s LSD test (FDR < 5%) was used to compare the three groups. *p < 0.05, **p < 0.01, ***p < 0.001

Furthermore, fold-change values (calculated as the ratio of the means between two groups) > 1.5 were taken into consideration. Using these screening criteria, 18 metabolites were identified; all were lipids and were elevated in INR relative to IR and control samples (Table 3). Acylcarnitines, including MC, LC, OC, SC, and PC had both high fold change and VIP values which could distinguish INR from IR and control samples. In addition, various fatty acids including polyunsaturated fatty acids and long chain fatty acids, also made contributions to distinguishing INR from controls. Metabolite set enrichment analysis showed that mitochondrial beta-oxidation of long chain saturated fatty acids and fatty acid metabolism were the main impaired pathways, which also suggest that acylcarnitines were the main differentiators between IR and INR (Fig. 2a). The levels of SC, MC, OC, PC and LC were not significantly different between control and IR samples, which suggest the mitochondrial beta-oxidation of IR samples were similar to control samples but were seriously impaired in INR samples (Fig. 2b).

**Table 3:** Metabolites with differing plasma levels among groups

Name	Pathway	Sub pathway	IR/control			INR/control			INR/IR		
			VIP	*p* value	FC	VIP	*p* value	FC	VIP	*p* value	FC
Myristoylcarnitine (MC)	Lipid	Fatty Acid Metabolism (Acyl Carnitine)	0.11	9.13E−01	1.06	1.81	4.12E−04	2.11	**2.13**	**1.97E−03**	**1.99**
Laurylcarnitine (LC)	Lipid	Fatty Acid Metabolism (Acyl Carnitine)	0.46	6.93E−01	1.2	1.69	7.29E−04	2.32	**2.27**	**6.16E−03**	**1.94**
Oleoylcarnitine (OC)	Lipid	Fatty Acid Metabolism (Acyl Carnitine)	0.68	4.34E−01	0.91	1.64	1.29E−03	1.74	**2.86**	**5.79E−04**	**1.9**
Stearoylcarnitine (SC)	Lipid	Fatty Acid Metabolism (Acyl Carnitine)	0.71	4.14E−01	0.9	1.71	8.77E−04	1.63	**2.80**	**3.73E−04**	**1.81**
Palmitoylcarnitine (PC)	Lipid	Fatty Acid Metabolism (Acyl Carnitine)	0.09	9.21E−01	1.03	1.64	1.83E−03	1.65	**2.54**	**4.21E−03**	**1.6**
2-hydroxydecanoate	Lipid	Fatty Acid, Monohydroxy	**2.07**	**8.42E−03**	**0.54**	1.62	5.35E−03	0.52	0.48	8.77E−01	0.95
10-nonadecenoate (19:1n9)	Lipid	Long Chain Fatty Acid	1.92	1.53E−02	1.88	**2.33**	**1.00E−05**	**2.70**	1.55	2.76E−02	1.44
Cis-vaccenate (18:1n7)	Lipid	Long Chain Fatty Acid	1.64	4.05E−02	1.36	**2.34**	**4.47E−06**	**1.90**	1.95	6.33E−03	1.4
Oleate (18:1n9)	Lipid	Long Chain Fatty Acid	1.71	2.08E−02	1.56	**2.37**	**1.80E−05**	**2.13**	1.63	3.15E−02	1.37
Margarate (17:0)	Lipid	Long Chain Fatty Acid	2.03	1.24E−02	1.49	**2.32**	**3.87E−06**	**1.92**	1.55	1.73E−02	1.28
Palmitate (16:0)	Lipid	Long Chain Fatty Acid	1.61	5.35E−02	1.38	**2.07**	**5.20E−05**	**1.76**	1.48	2.74E−02	1.27
Stearate (18:0)	Lipid	Long Chain Fatty Acid	1.44	1.14E−01	1.23	**2.19**	**8.39E−06**	**1.51**	1.96	3.19E−03	1.23
10-heptadecenoate (17:1n7)	Lipid	Long Chain Fatty Acid	**2.18**	**3.42E−03**	**2.16**	**2.21**	**7.44E−05**	**2.62**	0.95	2.43E−01	1.21
Palmitoleate (16:1n7)	Lipid	Long Chain Fatty Acid	**2.09**	**2.64E−03**	**2.35**	**2.11**	**1.61E−04**	**2.73**	0.80	3.88E−01	1.16
Dihomo-linoleate (20:2n6)	Lipid	Polyunsaturated Fatty Acid (n3 and n6)	1.68	1.06E−01	1.53	**2.11**	**2.27E−05**	**2.45**	1.63	7.34E−03	1.6
Epiandrosterone sulfate	Lipid	Steroid	**2.81**	**2.58E−05**	**0.28**	1.80	2.07E−04	0.40	0.77	5.41E−01	1.41
Androsterone sulfate	Lipid	Steroid	**2.36**	**4.13E−04**	**0.27**	1.58	1.41E−03	0.36	0.65	7.03E−01	1.35
Andro steroid monosulfate	Lipid	Steroid	**2.52**	**1.26E−03**	**2.84**	1.94	1.02E−03	2.88	0.12	9.48E−01	1.01

Features that meet all following conditions are highlighted using bold text: variable important in projection (VIP) values > 2, *p* values < 0.05 (FDR < 5%), or fold-change (FC) values > 1.5 or < 0.67

### Acylcarnitine levels were negatively correlated with CD4^+^ T cell count

**Fig. 3:**
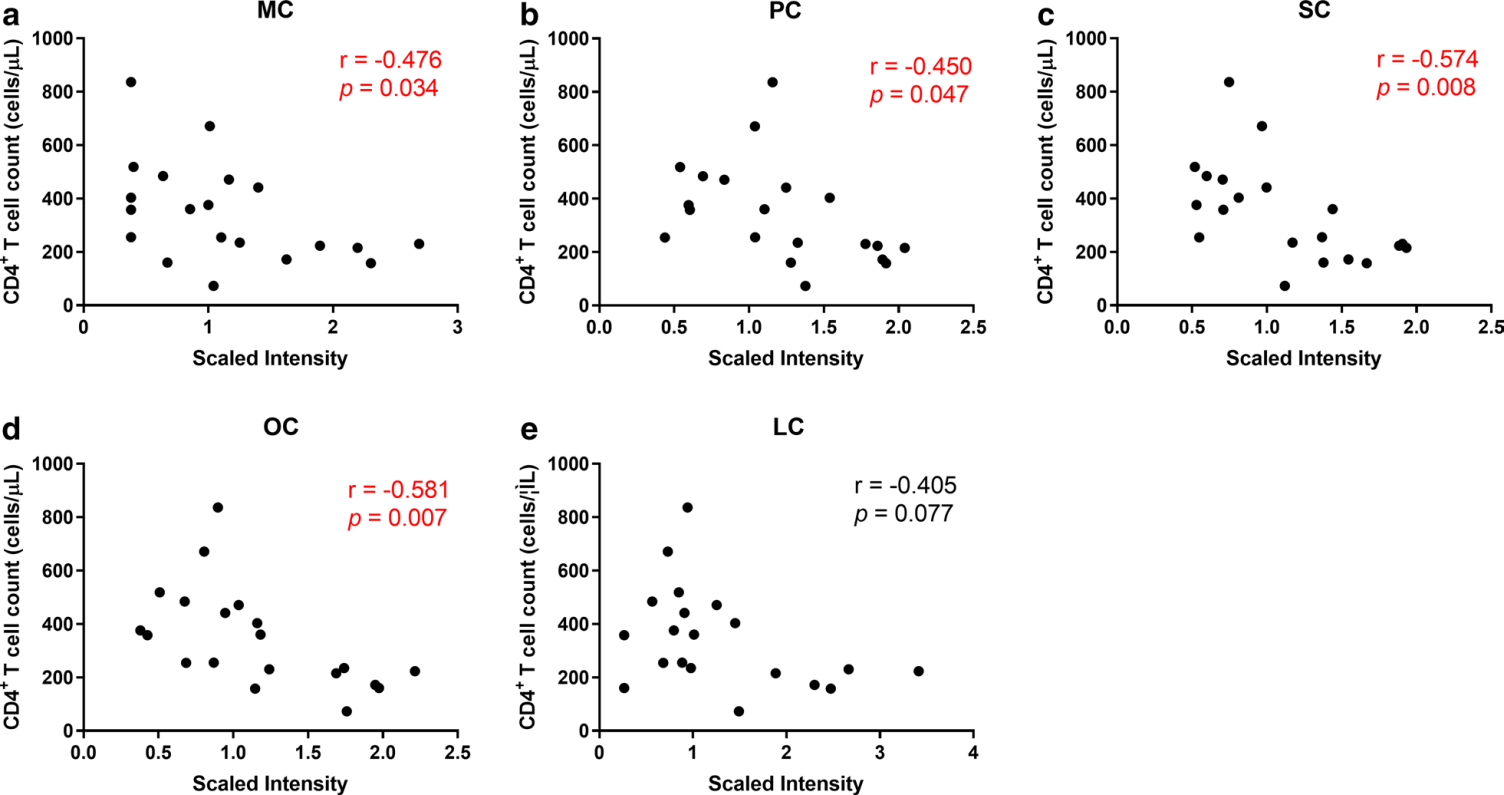
Correlations between metabolite levels and CD4^+^ T cell count. **a**–**e** The Spearman rank test was used to evaluate the correlation between acylcarnitines and CD4^+^ T cell count

Based on our finding that acylcarnitines were the main differentiators between INR and IR, we further explored the associations of acylcarnitines with immune recovery. We found that CD4^+^ T cell count in HIV-infected subjects were negatively correlated with MC (*p* = 0.034, r = -0.476; Fig. 3a), PC (*p* = 0.047, r = -0.45; Fig. 3b), SC (*p* = 0.008, r = -0.574; Fig. 3c), and OC (*p* = 0.007, r = -0.581; Fig. 3d), while LC was not significantly associated with CD4^+^ T cell count (*p* = 0.077, r = -0.406; Fig. 3e). These results indicate that elevated levels of MC, PC, SC, and OC in INR may be associated with decreased CD4^+^ T cell count.

### Levels of MC, PC, SC, and OC could be used to evaluate immune recovery

**Fig. 4:**
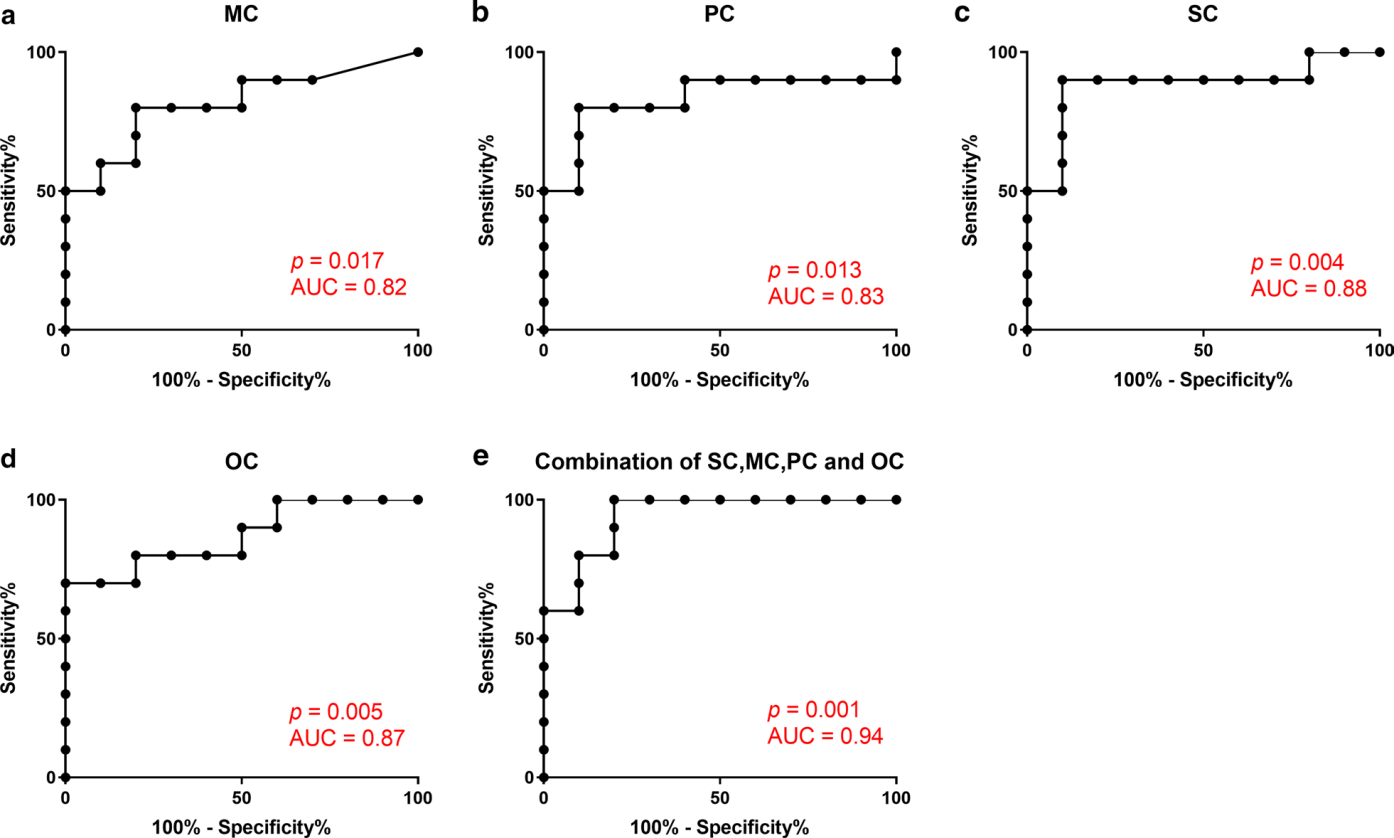
ROC analysis of acylcarnitine levels to distinguish INR and IR groups. **a**–**e** ROC analysis of the levels of MC, PC, SC, OC, or the combination of MC, PC, SC, and OC

Given the negative correlations between levels of acylcarnitines and CD4^+^ T cell count, we considered that these acylcarnitines were potential biomarkers for evaluation of immune recovery; therefore, we used ROC curves to evaluate the clinical value of these metabolites. The area under the curve (AUC) values for MC (*p* = 0.017; Fig. 4a), PC (*p* = 0.013; Fig. 4b), SC (*p* = 0.004; Fig. 4c), and OC (*p* = 0.005; Fig. 4d) were 0.82, 0.83, 0.88, and 0.87, respectively, indicating that these metabolites may be useful for evaluation of immune recovery. Next, we conducted ROC analysis of the four metabolites combined and the resulting AUC value was 0.94, suggesting that this combination of metabolites may be more meaningful for evaluation of immune recovery (*p* = 0.001; Fig. 4e).

### Levels of MC, PC, SC, and OC could predict CD4^+^ T cell count during long term ART

**Fig. 5:**
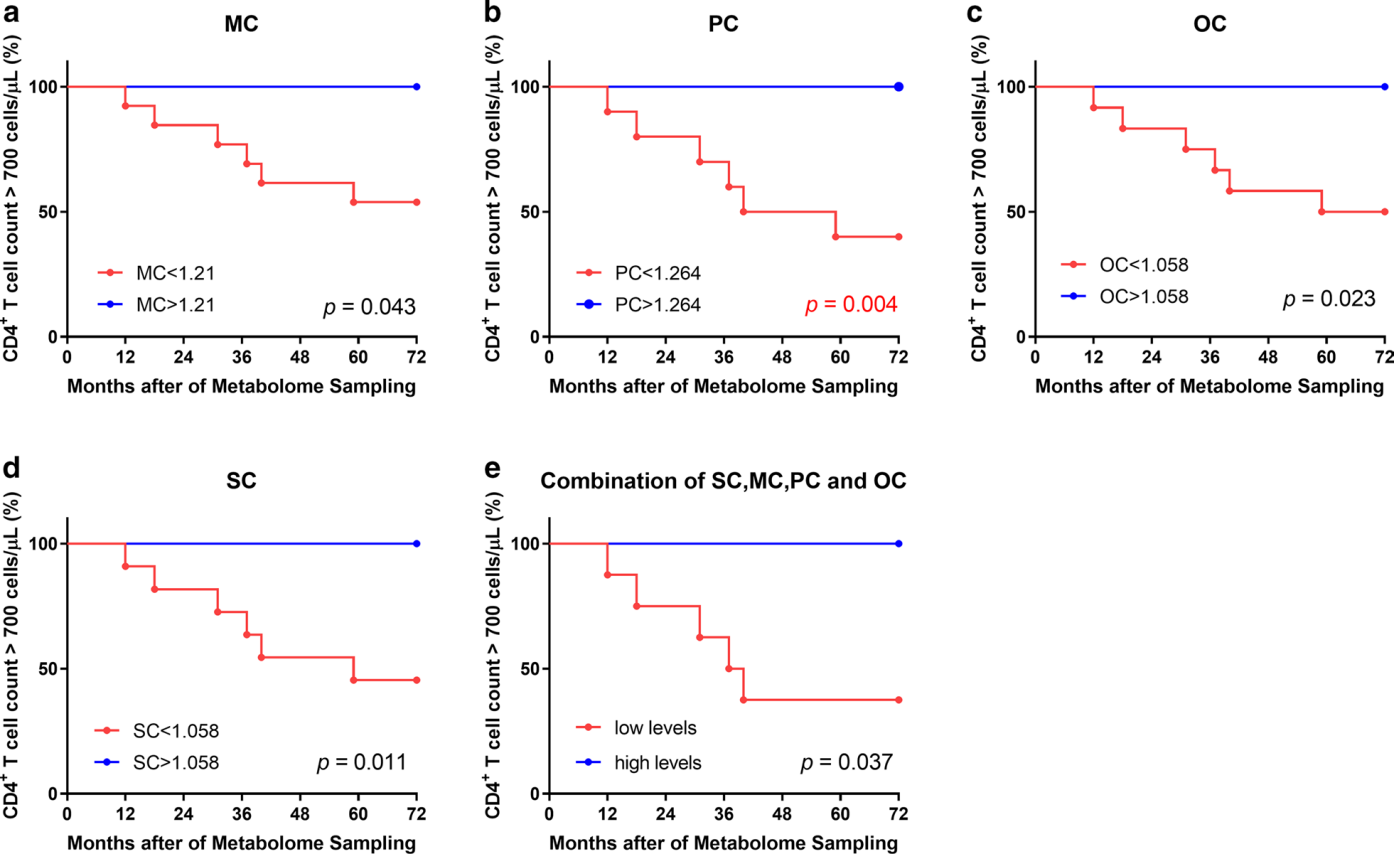
Analysis of the ability of acylcarnitine levels to predict CD4^+^ T cell count.** a**–**e** Survival analysis of levels of MC, PC, OC, SC, or the combination of MC, PC, SC, and OC. Subjects were divided into high-level and low-level groups, according to the cutoff points with the largest sum of sensitivity plus specificity in ROC curves. CD4^+^ T cell count of > 700 cells/μL were considered the end point. Kaplan–Meier survival curves and the log-rank test were used to assess predictive ability

To further validate these findings, we determined the cut-off point that generated from the largest sum of sensitivity plus specificity values in the ROC analysis, to regroup the 20 HIV-infected individuals, based on CD4^+^ T cell count data collected within 6 years after metabolome sampling (at 2 years after ART), and set CD4^+^ T cell count reaching 700 cells/μL as the outcome to conduct survival analysis. The results showed that MC (*p* = 0.043; Fig. 5a), PC (*p* = 0.004; Fig. 5b), OC (*p* = 0.023; Fig. 5c), and SC (*p* = 0.011; Fig. 5d) could help to predict CD4^+^ T cell count within 6 years. Moreover, we regrouped the samples according to levels of MC, PC, SC, and OC: samples with all metabolites at high levels were classified into the ‘high levels’ group, while samples with all metabolites at low levels were classified into the ‘low levels’ group. The results showed that analysis combining MC, PC, SC, and OC could predict CD4^+^ T cell count during long term ART (*p* = 0.037; Fig. 5e).

## Discussion

In this investigation, we analyzed metabolite profiles in plasma samples from controls, IR, and INR after 2 years of ART. We identified 18 metabolites that could distinguish the control, INR, and IR groups. The acylcarnitines, MC, PC, OC, and SC, were identified as the metabolites with the main profile differences between IR and INR and were associated with poor immune recovery of HIV-infected individuals receiving ART.

Our results indicate that lipid metabolites are the main differentiators that distinguish control, IR, and INR groups. HIV can disturb lipid and amino acid metabolism [18], while our data show that levels of lipid metabolites in HIV-infected patients are still higher than that of HIV- negative individuals. In addition, some ART drugs can affect lipid metabolism, probably by inhibiting the degradation of adipogenic transcription factors, such as SREBPs and PPAR-c [19–21]. All HIV-infected subjects in our study received NRTI combined with non-NRTI therapy for two years, and their viral loads were under the detection limit; therefore, the effect of the virus and drugs can be discounted, and the main cause of differences in lipid profiles can be attributed to immune recovery, in this study.

Further, we found that the fatty acid metabolites, acylcarnitines, were clearly elevated in INR. Acylcarnitines are synthesized by the combination of carnitine and acyl-CoA (activated fatty acid), and transported into the mitochondria by carnitine-acylcarnitine translocase [22]. Acylcarnitines are reconverted to acyl-CoAs with the help of carnitine-palmitoyltransferase-2 (CPT-2), and acyl-CoAs undergo β-oxidation in the mitochondrial matrix [23]. Increased levels of acylcarnitines are associated with reduced CPT-2 activity [24, 25], hence, the increased levels of acylcarnitines identified in the INR group in our study may indicate mitochondrial dysfunctional; for example, impaired translocase activity, which could result in the accumulation of acylcarnitines.

The association of high levels of acylcarnitines with poor immune recovery detected in this study may have been caused by CD4^+^ T cell apoptosis. PC can stimulate the activity of caspase-3/7 and caspase-8 to induce apoptosis of murine CD4^+^CD25^+^ T cells and the Jurkat cell line [26, 27]. Further, the combination of PC and carnitine can induce apoptosis of a colon cancer cell line, with O_2_^−^ generation during β-oxidation in the mitochondria [28]. Therefore, we speculate that PC may induce apoptosis of CD4^+^ T cells, by being absorbed by cells and then transported into mitochondria, where the increase in substrate results in O_2_^−^ accumulation, potentially causing apoptosis. In addition, there is evidence that MC or PC can activate JNK and ERK in proinflammation and stress signaling pathways in some murine cell lines [29, 30]. Acylcarnitines can also disrupt membrane barriers to solutes, leading to membrane solubilization [31, 32]. Hence, we hypothesize that long chain acylcarnitines may induce apoptosis of CD4^+^ T cells by promoting β-oxidation, leading to elevated oxidative stress, or membrane disruption; however, the specific mechanism underlying the induction of CD4^+^ T cell apoptosis by acylcarnitines remains to be explored.

It was reported that the accumulation of long-chain acylcarnitines in plasma were associated with liver or renal fibrosis in non-HIV infection[33–35], while the fibrosis was contributed to the loss of CD4^+^ T cells in HIV infection [36, 37]. Therefore, we postulated that acylcarnitines might associated with lymph node fibrosis and lead to poor immune recovery.

Our ROC analysis results demonstrate that MC, PC, SC, and OC can distinguish INR from IR, and the combination of these four metabolites exhibited higher sensitivity and specificity than any of them individually. Survival analysis also indicated that the combination of these four metabolites could be biomarkers for prediction of CD4^+^ T cell count to determine immune recovery in patients undergoing long term ART. Hence MC, PC, SC, and OC are important biomarkers of immune recovery and potential targets for immune intervention and treatments.

This study has some limitations. Although the age and BMI of the control, IR, and INR groups were matched, the data of lifestyle factors, diets and dietary supplements unavailable may have affected the results. We identified four metabolites which could be used to clearly distinguish INR from IR and predict CD4^+^ T cell count in patients undergoing long term ART; however, the results require verification in a future study with a larger sample size.

## Conclusion

In conclusion, we compared the metabolite profiles in plasma samples among control, IR, and INR groups in a Chinese cohort and found that MC, PC, SC, and OC were negatively correlated with CD4^+^ T cell count. The combined analysis of MC, PC, SC, and OC could be useful for indicating the immune status, and predicting immune recovery, during long term ART, potentially guiding clinical treatment decisions.

## Data Availability

The datasets used and/or analyzed during the current study are available from the corresponding author on reasonable request.

## Abbreviations

HIV: Human immunodeficiency virus
ART: Antiretroviral therapy
INR: Immunological non-responders
IR: Immunological responders
NRTI: Nucleoside reverse transcriptase inhibitors
UPLC-MS/MS: Ultra-high-performance liquid chromatography-tandem mass spectrometry
FC: Fold change
MC: Myristoylcarnitine
PC: Palmitoylcarnitine
SC: Stearoylcarnitine
OC: Oleoylcarnitine
ROC: Receiver operating characteristic
FDR: False discovery rate
OPLS-DA: Orthogonal partial least square discriminant analysis
VIP: Variable influence in projection
AUC: Area under curve
BMI: Body mass index
ESI: Electrospray ionization
RP: Reverse phase
